# Quad Zygoma: A Graftless Solution in Post-mucormycosis Maxillectomy

**DOI:** 10.7759/cureus.50014

**Published:** 2023-12-05

**Authors:** Arushi Beri, Sweta G Pisulkar, Bhushan P Mundada, Anjali Borle, Chinmayee Dahihandekar, Akansha Bansod

**Affiliations:** 1 Prosthodontics, Sharad Pawar Dental College and Hospital, Datta Meghe Institute of Higher Education and Research, Wardha, IND; 2 Oral and Maxillofacial Surgery, Sharad Pawar Dental College and Hospital, Datta Meghe Institute of Higher Education and Research, Wardha, IND

**Keywords:** hybrid prosthesis, tongue flaps, quad zygomatic implant, maxillectomy, mucormycosis

## Abstract

Mucormycosis, a fungal infection that commonly affects individuals with diabetes and compromised immune systems, often requires surgical excision and debridement. However, this can result in significant defects, posing a challenge for clinicians in terms of reconstruction and rehabilitation. Prostheses, local and regional pedicled flaps with or without bone grafts, and titanium mesh application are available options for maxillary reconstruction. Soft-tissue flaps are not sufficient to provide osseointegrated implants with both bone repair and structural support, which emphasises the quad zygoma's beneficial role in treating maxillary abnormalities. Patients benefit from quad zygoma, which uses zygomatic implants and eliminates the need for subsequent procedures, which shortens the course of treatment and lowers costs. Because zygomatic implants are securely fixed into the zygoma, temporary prostheses can be loaded right away. Then, four to six months later, a fixed prosthesis may be introduced. Clinical results with zygomatic implants often surpass those of bone grafting, representing a potential novel gold-standard approach for the compromised maxilla. This case report details the rehabilitation of post-mucormycosis patients with maxillary defects using quad zygomatic implants. The absence of complications during follow-up, conducted at 15, 30, 45, and 90 days, and subsequently monthly for two years, highlights the success of this approach. Evaluation parameters included soft tissue recovery, infection, wound separation, stability of prosthesis, eating effectiveness, and aesthetic outcomes. The positive outcomes observed at follow-up appointment emphasize the viability and effectiveness of quad zygomatic implants in addressing maxillary defects post-mucormycosis.

## Introduction

Maxillary reconstruction options encompass prostheses, local and regional pedicled flaps with or without bone grafts, and titanium mesh. Soft-tissue flaps lack bony reconstruction and structural support for osseointegrated implants, making the quad zygoma an advantageous choice for treating maxillary defects. It eliminates the need for adjunctive surgeries, reducing overall treatment duration and cost. Zygomatic implants are fixed into the zygoma and can be immediately loaded with a temporary prosthesis, followed by a fixed prosthesis after four to six months [1]. The innovative approach is considered a new gold-standard procedure for compromised maxillary bone. Follow-up evaluations assess healing, infection, dehiscence, prosthesis stability, eating efficiency, and aesthetics. Surgical excision and debridement frequently lead to defects of varying sizes, influencing functionality, appearance, morbidity, and overall quality of life. Among the available rehabilitation options for maxillectomy defects, the quad zygoma concept stands out. This method includes placing four zygomatic implants to rehabilitate individuals with inadequate bone height in the anterior and posterior maxilla [2]. Maxillary reconstruction after surgical interventions for mucormycosis poses challenges, with soft-tissue flaps often lacking sufficient support for osseointegrated implants. In addressing these issues, the quad zygoma technique, utilizing zygomatic implants, emerges as a promising solution. Zygomatic implants, fixed into the zygoma, provide stable support, eliminating the need for additional surgeries and significantly reducing overall treatment duration and cost. Immediate loading with a temporary prosthesis, followed by a fixed prosthesis after four to six months, enhances patient outcomes. This innovative approach, potentially establishing a new gold-standard procedure, offers a robust solution for compromised maxillary bone in severe atrophic cases. Follow-up evaluations assess healing, infection, dehiscence, prosthesis stability, eating efficiency, and aesthetics, emphasizing the viability and effectiveness of the quad zygoma approach [3-6].

## Case presentation

A male patient, age 32 years, sought medical attention at the outpatient department of Prosthodontics with a main issue that has persisted for the last two and half months: pus discharge from the upper maxillary region and loose teeth. The patient reported having foul-smelling nasal discharge, discomfort, and a persistent headache for the previous two to three weeks. The patient had a history of a prior COVID-19 infection, and a biopsy confirmed the diagnosis of mucormycosis. 

**Figure 1:**
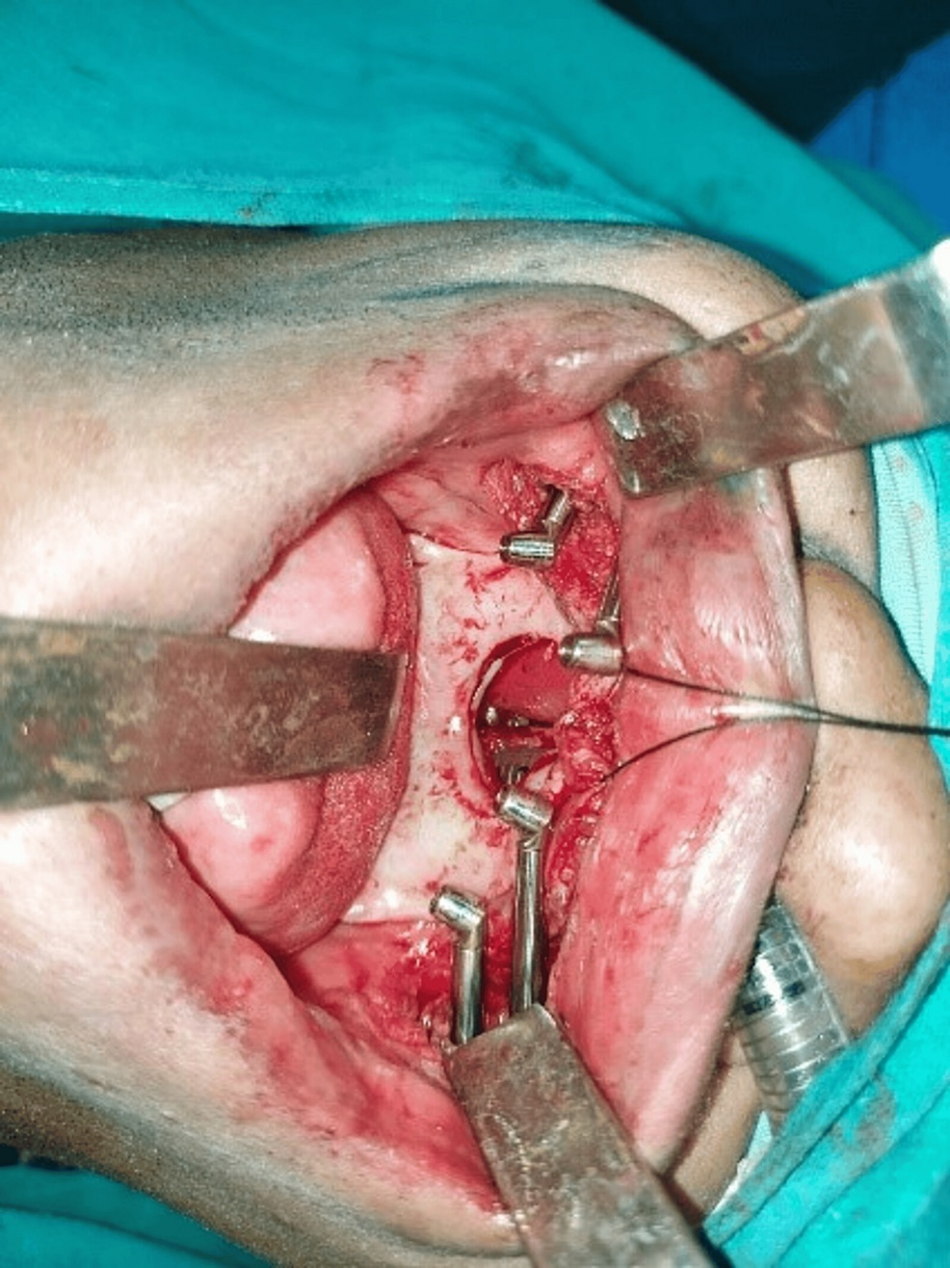
Four zygomatic implants placed at a 45-degree angulation following osteotomy

The patient underwent maxillectomy under general anesthesia, resulting in the establishment of intraoral communication. After this procedure, quad zygomatic implants were strategically placed to address the defect and facilitate the restoration of both function and esthetics. In the subsequent surgical phase, the patient, after undergoing comprehensive hematological investigations, received surgery under general anesthesia. The process included full-thickness mucoperiosteal flap elevation after bilateral crystal incisions in the maxillary arch. To reveal the zygomatic bone, subperiosteal dissection was also carried out labially and palatally. During the second stage of the operation, four 40 mm (1) and 50 mm (1) zygomatic implants were carefully positioned at a 45-degree angle after osteotomy, as shown in Figure 1.

**Figure 2:**
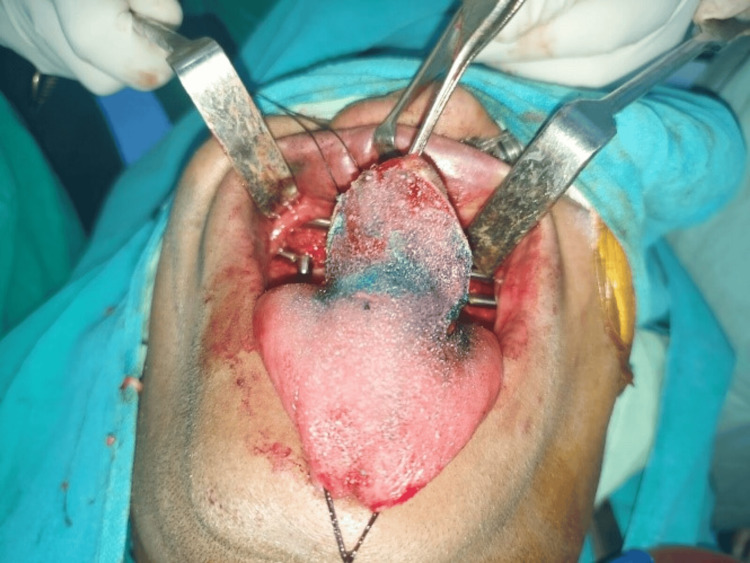
Tongue flap for closure of intra-oral communication

After successful hemostasis, Vicryl 3-0 (Ethicon, Inc., Raritan, New Jersey, United States) was used to complete the intraoral closure. A tongue flap was used to address intraoral communication (Figure 2).

**Figure 3:**
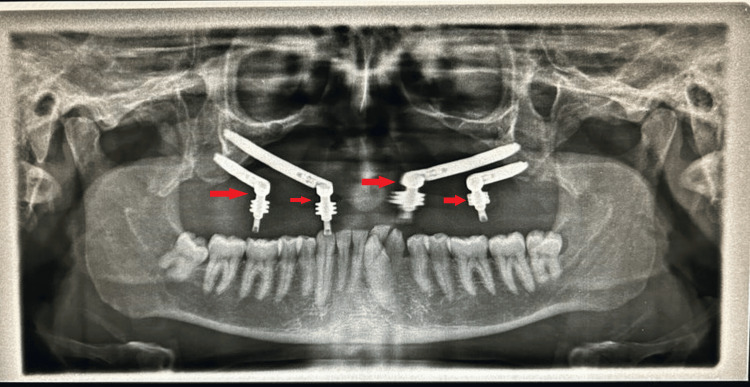
Orthopantomogram showing zygomatic implants Arrows are indicative of four zygomatic implants with abutments placed

The patient was recommended an antibiotic and analgesic regimen to last for one week. To evaluate the surgical result, an orthopantomogram (OPG) was performed, as shown in Figure 3.

Follow-up appointments were scheduled for one and two weeks following the procedure, and the one-month follow-up showed that the soft tissues had healed to their best potential. The postoperative instructions, including sinus care, were much the same as with any implant operation. Additional follow-up appointments at one and two weeks following surgery showed that the soft tissues were still mending optimally, and this was confirmed again at the one-month follow-up.

**Figure 4:**
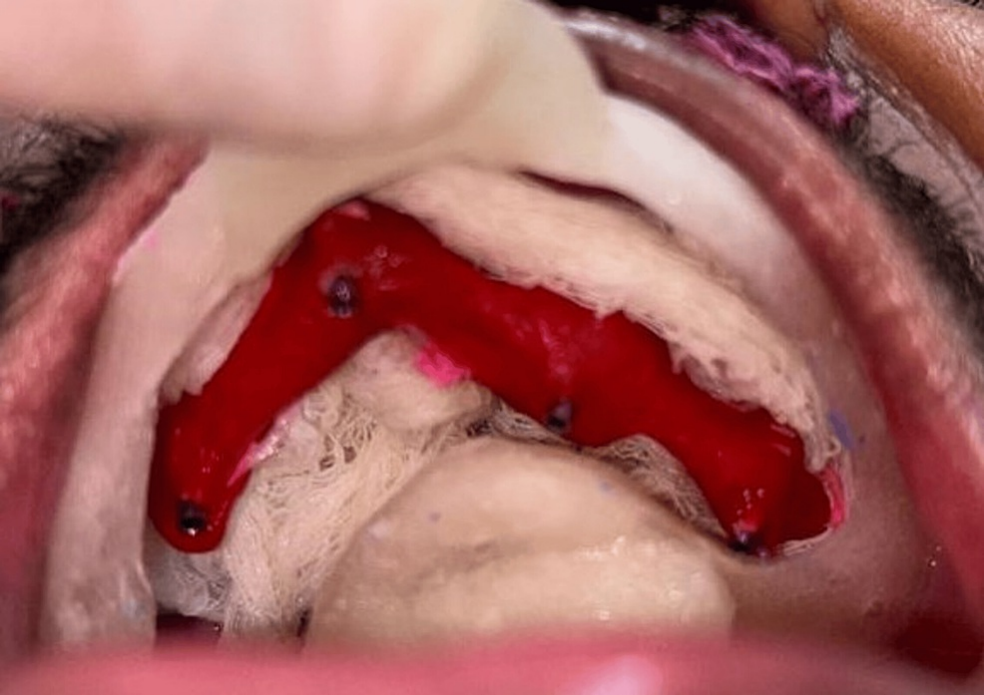
Open tray impression procedure

**Figure 5:**
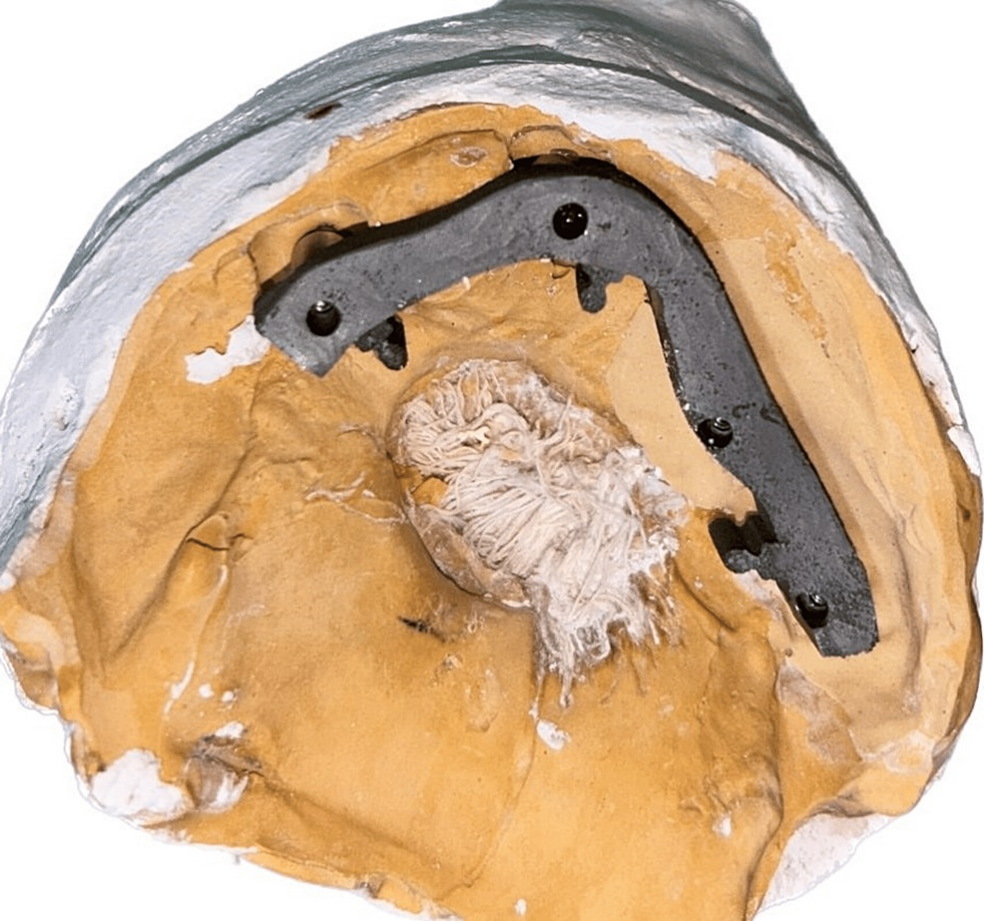
Metal framework seated on cast

**Figure 6:**
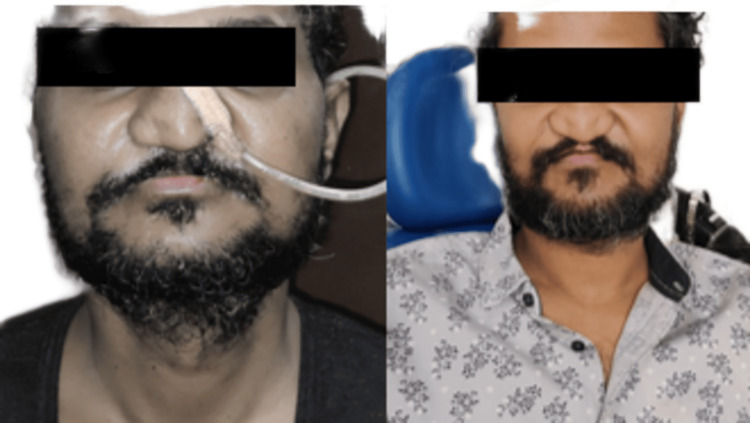
Comparison of pre and postoperative views

In the prosthetic phase, the patient was brought back three months later for the final prosthesis, and temporization was carried out three days following surgery. After creating an open tray impression (Figure 4), the metal framework was manufactured in the laboratory (Figure 5). A try-in was performed following the jaw relation procedure (Figure 6), and a hybrid prosthesis was supplied to close the defect. Figure 6 shows a comparison of pre and postoperative pictures.

## Discussion

Maxillary reconstruction has several objectives, including the restoration of the maxillary buttress, the separation of the oral and sino-nasal cavities, the achievement of appropriate midface projection, and the realization of the best possible esthetic result. Apart from that, goals include maintaining a patent nasal airway and establishing a functional dentition and occlusion. Clinicians investigate several reconstructive methods when confronted with obstacles such as advanced bone resorption or large maxillary sinuses that could hinder the feasibility of traditional implant procedures in the edentulous maxilla. There is a range of reconstructive treatments available, including microvascular techniques with free-tissue transfers, pedicled flaps, and maxillary prostheses [7-9]. Radial forearm, rectus abdominis, anterolateral thigh, scapular, fibula osteomyocutaneous, and iliac crest flaps are among the flaps in this array.

The fibula osteomyocutaneous flap is commonly used for maxillary reconstruction, particularly in cases where the maxillectomy defects are modest because it satisfies the particular needs for restricted bone width and volume of soft tissue [10]. Soft-tissue flaps alone have intrinsic limitations when it comes to providing structural support for osseointegrated implants and bone repair for maxillary deformities. In this case, the quad zygoma is an excellent therapeutic option for treating maxillary abnormalities. Implants positioned in the quad zygoma format may improve maxilla rehabilitation, despite the fact that it requires sophisticated surgical skills and is technique-sensitive [11-14]. While there are some possible dangers associated with zygomatic implants, such as tissue retraction, infection at the implant's tip, paraesthesia, and oral-antral communication, overall benefits usually outweigh these concerns. One noteworthy advantage of zygomatic implants is their ability to eliminate the need for additional procedures, which can drastically reduce the duration and cost of therapy overall. An expedient restoration of function and esthetics is made possible by zygomatic implants, which are anchored into the zygoma and provide immediate loading with a temporary prosthesis, followed by a fixed prosthesis after four to six months [15-22].

## Conclusions

Zygoma implants represent an essential alternative for providing stable support in cases when quick restoration with a microsurgical revascularized bone flap is impracticable following a maxillectomy. Zygoma implants are able to be placed even in cases where there are total deficiencies in the maxillary bone because of their design, which provides bicortical support through the malar bone. Compared to bone grafting, zygomatic implants have shown better clinical outcomes, which could establish a new gold-standard technique for maxillary bone that has been impaired.
